# Metformin: Multi-faceted protection against cancer

**DOI:** 10.18632/oncotarget.387

**Published:** 2011-12-24

**Authors:** Sonia Del Barco, Alejandro Vazquez-Martin, Sílvia Cufí, Cristina Oliveras-Ferraros, Joaquim Bosch-Barrera, Jorge Joven, Begoña Martin-Castillo, Javier A. Menendez

**Affiliations:** ^1^ Medical Oncology, Catalan Institute of Oncology, Girona (Catalonia, Spain); ^2^ Girona Biomedical Research Institute, Girona (Catalonia, Spain); ^3^ Unit of Translational Research, Catalan Institute of Oncology, Girona (Catalonia, Spain); ^4^ Centre de Recerca Biomèdica, Hospital Universitari Sant Joan de Reus, Institut d'Investigaciò Sanitària Pere Virgili, Universitat Rovira i Virgili, Reus (Catalonia, Spain); ^5^ Unit of Clinical Research, Catalan Institute of Oncology, Girona (Catalonia, Spain)

**Keywords:** Diabetes, cancer, metformin, cancer stem cells, senescence

## Abstract

The biguanide metformin, a widely used drug for the treatment of type 2 diabetes, may exert cancer chemopreventive effects by suppressing the transformative and hyperproliferative processes that initiate carcinogenesis. Metformin's molecular targets in cancer cells (e.g., mTOR, HER2) are similar to those currently being used for directed cancer therapy. However, metformin is nontoxic and might be extremely useful for enhancing treatment efficacy of mechanism-based and biologically targeted drugs. Here, we first revisit the epidemiological, preclinical, and clinical evidence from the last 5 years showing that metformin is a promising candidate for oncology therapeutics. Second, the anticancer effects of metformin by both direct (insulin-independent) and indirect (insulin-dependent) mechanisms are discussed in terms of metformin-targeted processes and the ontogenesis of cancer stem cells (CSC), including Epithelial-to-Mesenchymal Transition (EMT) and microRNAs-regulated dedifferentiation of CSCs. Finally, we present preliminary evidence that metformin may regulate cellular senescence, an innate safeguard against cellular immortalization. There are two main lines of evidence that suggest that metformin's primary target is the immortalizing step during tumorigenesis. First, metformin activates intracellular DNA damage response checkpoints. Second, metformin attenuates the anti-senescence effects of the ATP-generating glycolytic metabotype-the Warburg effect-, which is required for self-renewal and proliferation of CSCs. If metformin therapy presents an intrinsic barrier against tumorigenesis by lowering the threshold for stress-induced senescence, metformin therapeutic strategies may be pivotal for therapeutic intervention for cancer. Current and future clinical trials will elucidate whether metformin has the potential to be used in preventive and treatment settings as an adjuvant to current cancer therapeutics.

## DIABETES AND CANCER: FROM EPIDEMIOLOGY TO CELL BIOLOGY

In the relevant medical literature, type 2 diabetes has been linked to an increased risk of developing liver, pancreatic, colorectal, endometrial, kidney, urinary bladder and breast cancer as well as non-Hodgkin's lymphoma [1-4]. However, men with diabetes mellitus have a slightly lower risk of developing prostate cancer than average [5]. Moreover, the impact of type 2 diabetes on the development of cancer in diabetic patients and the specific survival of those patients have most likely been underestimated because an estimated 3-5% of the adult population is thought to have undiagnosed type 2 diabetes [6]. In a recent review and meta-analysis of all-cause mortality of patients with pre-existing diabetes, Peairs et al. [7] reported that patients with both breast cancer and diabetes had a significantly greater risk of death (i.e., 49%) compared with their nondiabetic counterparts. Although Peairs' study could be inaccurate due to the fact that over 40% of people age 20 or older have undiagnosed pre-diabetes or a delayed diagnosis of diabetes [8], Barone et al. [9] similarly observed a statistically significant increase in the risk of death in breast, endometrial, colon and rectal cancer patients who have diabetes compared with nondiabetic cancer patients. In newly diagnosed cancer patients, the prevalence of diabetes ranges from 8% to 18%, and diabetes is significantly associated with breast cancer in women [10], regardless of body mass. It should be acknowledged, however, that diabetes mellitus is not a homogeneous disease. Most studies on cancer patients have included patients with type 2 diabetes in part because of its high prevalence (i.e., 90% of all diabetes patients have type 2 diabetes) and because cancer is predominantly a disease of the elderly, a stage in life when type 2 diabetes is more frequent. Type 2 diabetes has metabolic and hormonal characteristics that differ from those in type 1 diabetes. Additionally, hyperglycemia and endogenous hyperinsulinemia can coexist for a long period, including during pre-diabetes. Importantly, strong evidence points toward insulin resistance and associated mitogenic hyperinsulinemia as a direct pathway connecting diabetes, obesity and metabolic syndrome with cancer.

## MOLECULAR LINKS BETWEEN DIABETES AND CANCER (I): INSULIN RECEPTORS

The membrane-bound receptor of insulin (IR) is a heterotetrameric protein that consists of four subunits; two subunits protrude from the cell surface and bind insulin, and the other two subunits span the membrane and protrude into the cytoplasm. Insulin binds to its receptor on the cell surface, causing a conformational change in the membrane-spanning subunits, which have tyrosine kinase activity. There are two isoforms of the IR, namely IR-A (also called fetal IR) and IR-B, which are produced by alternative splicing. The IR-A isoform is the predominant form in cancer cells because it can elicit mitogenic rather than metabolic effects [11]. Insulin-like growth factor 1 receptor (IGF-1R), which shares approximately 60% homology with IR, is a transmembrane tyrosine kinase activated by the binding of its ligand (IGF-1) and promotes mitogenic, metastatic, and anti-apoptotic phenotypes in breast cancer. The insulin and IGF-1 pathways are closely intertwined because both ligands can bind with different affinities the IR or the IGF-1R. Therefore, the activation of both receptors by insulin leads to the induction of two major intracellular transduction cascades, resulting in growth and enhanced survival. The survival pathway involves multiple anti-apoptotic targets of the phosphatidylinositol 3-kinase (PI-3K)/serine/threonine kinase AKT signaling pathway, and the proliferation pathway involves activation of the mitogen-activated kinases MEK and ERK [4].

## MOLECULAR LINKS BETWEEN DIABETES AND CANCER (II): INSULIN RESISTANCE AND METABOLIC SYNDROME

Insulin resistance in type 2 diabetic patients is a consequence, at least in part, of the upregulation of cytokines and free fatty acid derivatives that activate the inflammatory cascade and protein kinase C-zeta (PKC-zeta), a serine/threonine kinase that acts downstream of the PI-3K and insulin signaling pathways. PKC-zeta phosphorylates insulin receptor substrate-1 (IRS-1) and impairs the ability of IRS-1 to activate PI-3K in response to insulin [12]. Because PKC-zeta is located downstream of IRS-1 and PI-3K in established insulin signaling pathways, PKC-zeta may participate in a negative feedback pathway that promotes hyperglycemia. It should be noted that in contrast to type 1 diabetic patients, insulin resistance in patients with type 2 diabetes does not involve the “proliferation/mitogenic” pathway. As a consequence, the administration of exogenous insulin or insulin-mimetic compounds to type 2 diabetic patients might hyperactivate IR-driven mitogenic signaling, increasing the overall cancer risk in these patients. Activation of the IGF-1R by insulin can also significantly contribute to an increased risk of cancer in patients with type 2 diabetes. Additionally, obesity is linked with diabetes and cancer and increases the incidence of colon, esophageal and breast carcinomas. While type 2 diabetes is linked with obesity, which increases insulin resistance and accelerates diabetes progression [13, 14], insulin resistance leads to an enhanced sensitivity to the mitogenic effects of insulin, leading to a poorer prognosis in breast cancer patients.

Metabolic syndrome includes the following risk factors: high blood pressure, insulin resistance, obesity (especially abdominal obesity), and dyslipidemia. After following postmenopausal breast cancer patients who had not received chemotherapy, Pasanisi et al. [15] concluded that the risk of recurrence increased three-fold in women suffering from metabolic syndrome. Flanagan et al. [16] recently observed that metabolic syndrome was linked to a poor prognosis and overall survival in men with recurrent or newly diagnosed metastatic prostate cancer. Thus, metabolic syndrome is associated with a shorter time to the development of castration-resistant prostate cancer.

## METFORMIN: FROM DIABETES TO CANCER

### Anti-diabetic metformin: A definition

Metformin is a semi-synthetic biguanide with two methyl groups attached to the nitrogen nucleus of biguanide. This compound is derived from the hypoglycemic substance galegine, which is found naturally in goat's rue (*Galega officinalis*). Additionally, metformin is used to stimulate the activity of the mammary glands and is a diuretic used as an adjuvant for the treatment of diabetes. Metformin has been approved for use in the treatment of hyperglycemia, polycystic ovarian syndrome (PCOS) and metabolic syndrome. After oral administration, it is absorbed into the body within 1-3 hours, and 90% is eliminated by the renal system. Metformin decreases glucose absorption in the intestine and glucose production in the liver but does not stimulate insulin secretion. Consequently, it increases the uptake and utilization of glucose by skeletal muscle and adipose tissues. The lowering of blood glucose levels by metformin is only observed in people with diabetes and insulin resistance but has no effect on healthy people, except those who have been subjected to prolonged fasting. Metformin also increases the affinity of the insulin receptor for insulin, reduces hyperinsulinemia and improves insulin resistance. Several days after administering the drug, insulin levels are reduced by 25-33% in both diabetic and nondiabetic patients. Metformin can also decrease fatty acid uptake and oxidation in skeletal muscle cells while lowering circulating levels of total cholesterol, Low-density lipoprotein (LDL) and triglycerides. Additionally, in contrast to sulfonylureas, metformin promotes weight loss (approximately 2 kg), and once weight loss is achieved, weight loss is maintained. Generally, metformin is well tolerated, with only 5% of patients being intolerant. Its side effects are mild and reversible gastrointestinal disorders (30%); metallic taste (3%), which is reversible with continued use, and decreased levels of B12 in 6% of patients after 29 weeks. Metformin is a safe drug with a low risk of lactic acidosis, which affects 3 out of 100,000 people per year (50% of which are fatal). Renal failure, congestive heart failure and an age greater than 80 years are factors associated with the highest risk group.

**Figure 1:**
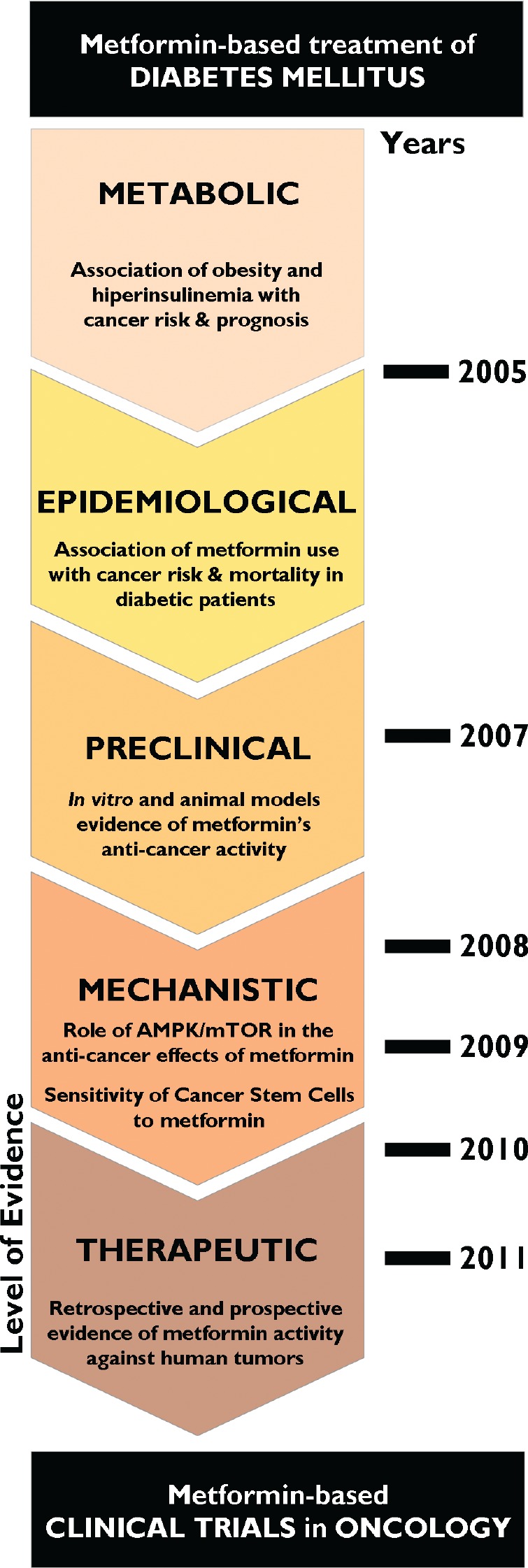
Metformin and cancer: From phenomenology to molecular understanding in less than a decade Since an initial report by Evans et al. [22] revealing that metformin use in people with type 2 diabetes was associated with reduced cancer incidence, the hypothesis that metformin may have clinically relevant preventive and treatment effects in human cancer exploded as an ever-growing research field, as scientists discovered mechanistic connections to pivotal cancer markers and even cancer stem cells. Amazingly, the molecular and clinical breakthroughs in metformin and cancer have taken place during only the past decade.

Between June 2010 and June 2011, 31 reviews were indexed in *PubMed* on “metformin and cancer.” A recent review published in *Clinical Science* discussed the implications of hyperinsulinemia and insulin resistance in the development and progression of cancer [17]. Here, we will report the most relevant publications supporting the potential benefit of metformin as a novel multi-faceted drug to prevent and treat cancer. These studies justify the need for clinical trials to confirm the potential anti-neoplastic activity of metformin, as the preclinical and clinical evidence currently available for standard practice is largely lacking (Fig. 1).

### Metformin and cancer: Epidemiological evidence

Many studies have found a lower incidence of and mortality from cancer in diabetic patients who have been treated with metformin. Examples include the large, retrospective study by Bowler et al. [18] that involved patients who had been treated with either metformin or sulfonylurea for a period of at least 5 to 5.5 years. This group determined that metformin was associated with lower overall mortality (3.5% versus 4.9% for the sulfonylurea cohort) and mortality as a result of cancer (an HR of 1 for metformin and no insulin use versus an HR of 1.3 (CI 1.1-1.6) for the sulfonylurea group). A Dutch labor prospective observational trial recently followed patients who had used metformin and other anti-diabetic agents for 9.6 years [19]. In total, 1300 patients were followed; 289 had been treated with metformin, and 1064 had been treated with another drug. This group found that metformin use was associated with lower mortality due to cancer with an adjusted hazard ratio of 0.43 (95% CI 0.23-0.80), and this association was dose-dependent, resulting in a 42% probability of mortality due to cancer for every 1 g increase in the metformin dose. Additionally, Currie et al. [20] observed a decrease in the diagnosis of cancer in patients taking insulin and metformin. Another meta-analysis found a 31% reduction in the cancer rate in patients taking metformin and found that this relationship was also dose-dependent [21]. In a retrospective study of type 2 diabetic patients, Evans et al. [22] found reduced cancer rates in patients who had been treated with metformin, and this relationship was again dose-dependent. In this study, the cancer incidence decreased more than 50% (adjusted odds ratio of 0.56; 95% CI of 0.43-0.74) if the patients had been treated with metformin for more than 4 years.

The treatment of type 2 diabetic patients with metformin has also been reported to lower mortality due to several solid tumor types. Although little is known about the effect of metformin on human colorectal carcinogenesis, recent epidemiological studies have shown reduced incidence of colorectal cancer in patients with type 2 diabetes taking metformin when compared with those patients who do not take metformin [20, 23-25]. Patients with colorectal and pancreatic carcinomas who had been treated with metformin showed a 30% improvement in survival when compared with patients who had been treated with other anti-diabetic treatments [25-27]. In patients with hepatocellular carcinomas who had been treated with radiofrequency, diabetes was associated with lower survival rates versus nondiabetic patients, but metformin users had better survival outcomes (adjusted hazard ratio of 0.24; 95% CI of 0.07-0.80) [28]. More importantly, treatment with drugs other than metformin and a tumor size larger than 2.5 cm were independent variables associated with lower survival rates in the entire studied population. Early epidemiological studies suggested an inverse relationship between diabetes and prostate cancer [5, 29, 30]. Accordingly, a 44% risk reduction of prostate cancer incidence in Caucasian men on metformin therapy has been reported in a population-based case-control study [31]. However, Patel et al. [32] recently reported that metformin use does not have a significant beneficial effect after prostate cancer diagnosis.

Recently, a retrospective study published by Jiralerspong et al. [33] showed an increase in the effectiveness of neoadjuvant chemotherapy in breast cancer patients who took metformin concomitantly with systemic therapy in patients with and without diabetes. Specifically, diabetic patients who had been treated with metformin had a pathologic complete response (pCR) rate three times higher (24%) than those who had not been treated with metformin (8%). Importantly, the rate of pCR in patients without diabetes was 16%. Although metformin use has been shown to associate with a decreased risk of breast cancer in the long term [34, 35], the overall survival rate was the same as in nondiabetic women despite a higher pCR rate in the metformin-treated group [33]. Bayraktar et al. [36] observed that triple negative breast cancer patients who did not receive adjuvant metformin and nondiabetic patients tended to have a higher risk of distant metastases compared with the metformin-treated group. Their findings, however, suggest that metformin use during adjuvant chemotherapy does not significantly impact survival outcomes in diabetic patients with highly aggressive triple receptor-negative breast cancer. In the first line of chemotherapy treatment for advanced lung cancer, diabetic patients who were being treated with metformin demonstrated better overall survival and longer progression-free survival and disease control [37]. Although diabetes is associated with a lower incidence of prostate cancer, mortality from prostate cancer is higher in type 2 diabetic patients [38]. Recently, the MD Anderson Cancer Center presented a retrospective study of 233 diabetic patients with prostate cancer. This multivariate analysis analyzed obesity, PSA, grade, age, and use of diabetes drugs and showed that treatment with thiazolidinedione and metformin were significant predictors of improved survival (HR: 0.45 and 95% CI: 0.21-0.9; HR: 0.55 and 95% CI: 0.31-0.96, respectively) [39].

### Anti-tumor effects of metformin: Molecular mechanisms

Although the abovementioned epidemiological studies have provided relatively consistent results suggesting that people with type 2 diabetes receiving metformin demonstrate a lower risk and improved outcomes with most common cancers, caution is needed when directly translating these findings to clinical cancer prevention and treatment. Most of these studies were retrospective, clinical, or hospital based and, therefore, susceptible to selection bias. For instance, several studies did not exclude individuals with prior cancers; moreover, patients who received metformin significantly differed in many key factors from those who did not receive the drug closely related to cancer risk, including age, obesity, and smoking history. Despite these methodological limitations, both the epidemiological findings and the suspected anti-cancer effects of metformin have contributed to the interesting hypothesis that metformin may exert clinically relevant effects in the primary and secondary prevention of human carcinomas. The anti-cancer effects of metformin based upon its dual action on systemic insulinemia (i.e., maintaining glucose levels and insulin at physiological levels in the plasma) and its direct, targeted action against cancer cells (with pleiotropic inhibitory effects on multiple pathways involved in survival and metastasis) [40] need to be further studied.

### Metformin and the ATM/LKB1/AMPK axis

Metformin largely exerts its effects by activating 5' adenosine monophosphate (AMP)-activated protein kinase (AMPK). AMPK is a major metabolic sensor involved in regulating cellular energy homeostasis. In conditions of cellular stress (e.g., glucose deprivation, hypoxia, oxidative stress, or ischemia), the ratio of AMP/ATP increases, which induces the activation of AMPK. Once activated, AMPK inhibits anabolic processes that require energy and activates catabolic processes that produce energy. The activation of AMPK is mediated by other proteins including the enzymes LKB1 (i.e., the serine-threonine kinase STK11), CaMKK (calcium/calmodulin-dependent protein kinase) and TAK1 (TGF-β-activated protein kinase 1) [41-44]. Indeed, the tumor suppressor LKB1 (one of the most commonly mutated genes in lung and pancreatic cancers and melanomas) can mediate the action of metformin on AMPK activity [45-47]. The absence or decreased expression of LKB1 in human breast carcinomas is associated with poor prognosis [48], which suggests that the inhibition of tumorigenesis by metformin may depend on the status of LKB1 [49]. However, a recent study in mice found that hyperinsulinemia-mediated loss and/or mutation of LKB1 is a predictor of sensitivity to metformin [50].

A polymorphism in the *LKB1* gene is associated with ovulatory response to treatment of Polycystic Ovarian Syndrome (PCOS) patients with metformin alone in a prospective randomized trial [51]. Additionally, genetic polymorphisms in the cell surface transporter organic cation transporter 1 (OCT1), which is required for the efficient action of metformin, have been shown to underlie metformin resistance in some patients with type 2 diabetes and PCOS [52-54]. Although it is likely that *OCT* gene polymorphisms may significantly affect the efficacy and toxicity of metformin against human cancer cells [55], this remains to be confirmed. Recently, a region in the *ATM* gene (ataxia telangiectasia, mutated) that modulates the response to metformin in type 2 diabetic patients was discovered [56-58]. *ATM* is a tumor suppressor gene implicated in DNA repair and cell cycle control. The authors of this study concluded that ATM is required for the full anti-glycemic activity of metformin. ATM phosphorylates LKB1 and other components of the insulin pathway but can also modulate the activation status of AMPK independent of LKB1 [59-61]. This suggests that an unexpected relationship with DNA repair pathways may explain, at least in part, metformin's efficacy against cancer cells. Using cultured tumor cells, we recently confirmed that metformin promotes activation of ATM and ATM targets, such as the protein kinase Chk2, suggesting a causal linkage between metformin's mechanism of action and metformin's cancer preventative effects [62].

### Metformin and endogenous lipogenesis

One of the consequences of AMPK activation is the inhibition of lipogenesis in malignant lesions. Tumor cells require high levels of *de novo* fatty acid synthesis, and the most aggressive phenotypes of breast cancer have a high rate of lipid metabolism also dependent on and involved in proliferation and cell survival [63-65]. Furthermore, tumor induction by certain oncogenes causes the activation and expression of enzymes for the *de novo* synthesis of fatty acids such as acetyl-CoA carboxylase (ACC) and fatty acid synthase (FASN). Notably, inhibition of exacerbated lipogenic metabolism in tumor cells might result in inhibition of the activity and expression of upstream oncoproteins [66-68]. The effects of metformin on energy homeostasis in normal and cancer cells have been characterized by the blocked activation or expression of key fatty acid biosynthesis enzymes (e.g., ACC, FASN, HMGCR) and enhanced expression of regulators of mitochondrial biogenesis (e.g., PCG-1α) [69-74]. Together, these effects on energy homeostasis are expected to significantly contribute to the antiproliferative activity of metformin by inhibiting endogenous fatty acid biosynthesis and shifting cellular bioenergetics to catabolism. Such changes in lipid metabolism have been demonstrated experimentally using several FASN and ACC blockers to inhibit growth and induce apoptotic cell death in cancer cells [75-77].

### Metformin and mammalian target of rapamycin (mTOR)

mTOR is involved in regulating cellular energy homeostasis by modulating the activity of different cellular processes such as protein synthesis and autophagy [78-80]. mTOR plays a critical role in cell growth and tumorigenesis in different tumors, and its activation correlates with cancer progression, adverse prognosis and resistance to chemotherapy and molecularly-targeted therapies [81-84]. mTOR activation occurs frequently in breast cancer and results in a poorer prognosis. The activated form of AMPK inhibits mTOR activity via the phosphorylation and stabilization of the tumor suppressor tuberous sclerosis complex 2 (TCS2). As such, metformin inhibits the mTOR-signaling pathway in an AMPK-dependent manner, which may provide an explanation of the observed anti-neoplastic actions in breast cancer [85-88]. In other tumors where mTOR plays an important role, including renal cell carcinomas, a mechanism by which metformin inhibits tumorigenicity via mTOR and activation of AMPK has been demonstrated [89]. Based on these data, a combination of therapies directed against AMPK and the PI3K/AKT/mTOR pathways has emerged as an option for the treatment of cancer [82, 90]. The combination treatment of temsirolimus and metformin in a phase I clinical trial on solid tumors was feasible with only one grade 1 hyperglycemia case [91]. It should be noted that mTOR inhibition might also occur in the absence of AMPK activation, for example, by inhibiting IGF1, the insulin receptor and AKT [92]. Therefore, metformin can inhibit mTOR by decreasing the levels of insulin or IGF1 independent of AMPK.

### Metformin and estradiol

Active AMPK inhibits the expression of the aromatase gene in breast adipose tissue by decreasing the local production of estrogen [93]. Furthermore, obese patients with breast cancer exhibit a higher expression of this gene and higher levels of estrogen in breast tissue as a result of increased plasma leptin synthesis, which is caused by obesity. Leptin inhibits AMPK by increasing the expression of aromatase; however, adiponectin activates AMPK. Therefore, metformin-based treatments aimed at activating AMPK and restoring the leptin/adiponectin axis may decrease the occurrence of breast cancer in obese patients.

### Metformin and the mitotic cell cycle

AMPK is involved in the process of cell division. Recent studies from our laboratory have shown that the phosphorylated form of AMPK has a space-time dynamic during mitosis, as it is located at the centrioles during the initial stages and in the constriction ring during the final stages of mitosis [94-96]. Metformin also decreases the expression of many genes involved in mitosis including kinesins, tubulins, histones, auroras and polo-like kinases [97]. Therefore, the chronic activation of AMPK by metformin alters mitosis, and the severity of these changes may depend on the status of p53 [98]. Specifically, chronic activation of AMPK leads to the activation of p53 and cellular senescence. Inhibiting oxidative phosphorylation with metformin has been suggested to increase glycolysis and autophagy in cells bearing native p53. Conversely, cancer cells with a mutated p53 that have been treated with metformin are unable to reprogram metabolism, and the cell undergoes apoptosis [99].

### Metformin and apoptosis: Impact on chemotherapeutic efficacy

Metformin increases the cytotoxicity of some drugs. In breast and lung cancer cell lines, metformin and paclitaxel synergistically induce cell cycle arrest, and the combination increases the number of cells in the G_2_/M phase, inhibiting tumor cell proliferation independent of LKB1 [100]. Additionally, in ovarian cancer, metformin inhibits tumor growth in nude mice in a dose-dependent manner and reduces the number of lung metastases, proliferation (determined by Ki-67), vascular density and angiogenesis as measured by VEGF [101]. More importantly, the effects were synergistic with cisplatin treatment. Lliopoulos et al. [102] also demonstrated a synergistic antitumor effect of metformin with cisplatin, doxorubicin and paclitaxel in an animal model. Additionally, metformin induces both caspase-dependent and poly (ADP-ribose) polymerase-dependent cell death in breast cancer cells [103].

### Metformin and oncogenes: Impact on targeted cancer therapy

Metformin treatment has been shown to efficiently inhibit endogenous initiation and progression of spontaneous mammary tumors in *HER2*-transgenic mice [104, 105]. Additionally, metformin decreases the levels of HER2 activity and expression in cell lines in a dose-dependent manner. At low doses (in the micromolar range), the HER2 tyrosine kinase activity is blocked, but expression levels are not affected [87]. At higher concentrations (in the millimolar range), the expression of HER2 protein is downregulated [106]. Furthermore, the metformin-induced inhibition of HER2 is independent of the molecular mechanism that contributes to the overexpression of HER2 (i.e., gene amplification or transcriptional activation) [106]. Metformin has different effects on gene expression depending on whether a cell line is HER2-positive or HER2-negative [97]. In HER2-negative breast cancer cell lines, the expression of genes related to mitosis is decreased in response to metformin. Meanwhile, in HER2-positive breast cancer cell lines treated with metformin, genes involved in apoptosis are overexpressed. Although ongoing clinical-translational research is required to provide evidence for the usefulness of therapeutically combining metformin with HER2-targeted therapies [107], it is worth mentioning that metformin acts synergistically with the anti-HER2 monoclonal antibody trastuzumab (Herceptin™) to eliminate stem/progenitor cell populations in *HER2*-gene amplified breast carcinoma cells growing as mammospheres [108]. Moreover, metformin treatment efficiently reverses secondary resistance of HER2-overexpressing cancer cells to the dual HER1/HER2 tyrosine kinase inhibitor lapatinib (Tykerb™) by suppressing pro-survival pathways (i.e., the anti-apoptotic protein survivin) [109-111].

In HER2-positive tumors, metformin has a dual effect: 1) inhibiting the activity/expression of the HER2 onco-tyrosine kinase and 2) blocking the action of mTOR, a possible mechanism of resistance to trastuzumab [88, 112]. Additionally, by decreasing the levels of circulating insulin and IGF, activation of the IGFR pathway will be avoided. Because transactivation of the IGFR pathway is a well-recognized mechanism underlying *de novo* (i.e., primary) [113] and acquired (i.e., secondary) [114, 115] resistance to anti-HER2 therapies, metformin's ability to simultaneously target HER2, while preventing increased IGF-IR signaling may represent a potential therapeutic tool in breast carcinomas resistant to HER2-directed therapy. In support of this, a recently conducted pre-clinical study with trastuzumab-sensitive parental breast cancer cell lines (i.e., BT474 and SKBR3) and trastuzumab-resistant breast cancer sublines (i.e., BT-474-HR20 and SKBR3-pool2) showed that metformin treatment causes significantly more inhibition of proliferation and clonogenicity in trastuzumab-resistant sublines via disruption of HER2/IGF-IR complexes (which are solely present in the resistant sublines) [116]. Importantly, this effect occurred without altering HER2 expression or reduction of IGF-IR expression or activity in the trastuzumab-resistant but not in the sensitive breast cancer cells. The activity of AMPK in cardiac cells is associated with stress-induced survival in response to cytokines or energy depletion. Thus, the concurrent blockage of oncogenic receptors such as HER2, while activating AMPK-related catabolic pathways with metformin would be a highly efficacious therapy to prevent, delay and/or reverse resistance to the HER2 inhibitor while decreasing the risk of cardiomyopathy [109, 117].

## METFORMIN: FROM EPIDEMIOLOGICAL AND PRE-CLINICAL EVIDENCE TO CANCER PREVENTION

The ever-growing amount of epidemiological evidence on the relationship between metformin usage and cancer incidence and mortality among diabetics does not necessarily imply a universal chemoprevention effect of metformin, especially because it may not have significant (if any) effects in nondiabetics. We should acknowledge that population studies have mostly been retrospective and confined to diabetic patients, in whom factors that are relatively unimportant in the general population may have significant roles [118]. Indeed, there is still a lack of retrospective clinical evidence for antitumor activity of metformin in nondiabetic patients. Because an ideal strategy for cancer prevention should employ a limited course of low-toxicity therapy to suppress premalignant lesions in high-risk cancer patients, forthcoming studies should focus on the clinical application of metformin and/or other metformin-related biguanides as suppressors of premalignant lesions in a broad spectrum of tissues. Metformin-based, large-scale cancer prevention trials would be more justifiable with strict criteria specifying high-risk populations in which metformin is expected to provide a greater clinical benefit [119, 120]. Metformin's ability to increase the mean lifespan of tumor-free mice while simultaneously decreasing the risk of age-related death underscores its ability to reduce cancer incidence among type 2 diabetics. Evans et al. [22] reported that the risk of subsequent cancer diagnosis was significantly reduced in patients with type 2 diabetes receiving metformin and that metformin's protective effect was increased with metformin use (i.e., dose and/or time of metformin treatment). Furthermore, a recently conducted retrospective study reported an impressive 56% decrease in the risk of breast cancer among diabetics receiving metformin when compared with diabetics being treated with other anti-diabetic therapies [121]. In addition, available data have revealed that reductions in cancer mortality related to metformin use are similar in magnitude to reductions in cancer incidence, suggesting that the anti-cancer effects of metformin largely depend on (or are restricted to) its preventive effects [119].

### Mechanisms of metformin cancer prevention (I): Cancer stem cells

Because high levels of IGF-1 and estradiol can favor the generation and/or maintenance of mammary tissue-specific stem cells [122], pharmacological measures to enhance insulin sensitivity (e.g., metformin) might significantly reduce the risk of metastatic progression in premalignant lesions. Breast stem cell niches not only support the self-renewal and maintenance of stem cell identity but also control stem cell number and proliferation [123]. If regulators of niches also function as mitogens for the reservoir of undetected, pre-invasive breast cancer lesions and/or dormant cancer stem cells [CSCs] within premalignant lesions *in situ* [124, 125]), then metformin's ability to decrease systemic metabolic biomarkers including insulin, IGF-1 and estradiol would regulate generation and/or maintenance of mammary stem cells and/or their niches. As such, this would regulate the number and proliferation rate of tumor progenitors residing at premalignant lesions. Evidence for this CSC-centered hypothesis underlying breast cancer prevention by metformin is the observation of increased breast cancer risk within 2 years in diabetic women receiving the insulin analog glargine [126-128]. Hormonal factors influencing the natural history of breast cancer (e.g., insulin, IGF-1, estradiol) could have effects in very short amounts of time. Therefore, the increased breast cancer risk in glargine users likely reflects the growth of subclinical malignant lesions to clinically diagnosable volumes rather than the initiation of new tumors [129]. As such, metformin's inhibitory effects on CSC-like subpopulations in intraepithelial neoplasias might prevent invasive carcinomas including breast cancer in pre- and post-menopausal women [120, 130].

The theory of CSCs (also called tumor-initiating cells) suggests that tumors consist of two cell subpopulations. CSCs have the capacity to self-renew while giving rise to other distinct phenotypic subpopulations upon differentiation, which contributes to cellular heterogeneity within human tumors [131-133]. Presumably, tumor relapse is due to the intrinsic chemoresistance of CSCs. Therefore, drugs that attack both the tumorigenic subpopulation of CSCs and the more differentiated and proliferating population could efficiently prevent disease recurrence [134, 135]. A landmark study that demonstrated the effect of metformin on CSCs was conducted by Hirsch et al. [136] using mice with a human breast cancer xenograft. Metformin treatment was found to specifically eliminate CD44^+^/CD24^−/low^ CSCs and had a synergistic effect with doxorubicin, which resulted in reduced tumor burden and delayed tumor recurrence and was more effective than either agent alone [136]. Furthermore, metformin combined with doxorubicin/cisplatin or paclitaxel delayed tumor relapse better than either agent alone. Surprisingly, the combination was effective with a four-fold lower dose of doxorubicin, thus decreasing the toxicity of chemotherapy and improving its efficiency. In this case, metformin could be preventing the dedifferentiation of tumor cells to CSCs [102].

Given that metformin appears to act on a subpopulation of tumor stem cells, it seems reasonable that more aggressive breast cancer subtypes enriched with CSC-like features, such as basal-like (triple negative) and HER2 cells, would be more sensitive to the action of metformin [88]. Recently, we found that proliferation and size of CSC multicellular “microtumors” (i.e., mammospheres) in non-adherent and non-differentiating conditions were inhibited by metformin, indirectly reflecting metformin's ability to suppress stem cell renewal and progenitor cell proliferation, respectively [108]. Perhaps more importantly, metformin treatment appears to significantly alter the genetic and/or epigenetic plasticity of CSCs because it can resensitize mammosphere-initiating cells to HER2-targeted drugs [108]. Furthermore, we recently confirmed that among the different molecular classes of breast cancer, triple-negative/basal-like breast cancer cells are significantly more sensitive to the growth-inhibitory effects of metformin [137-139]. In the triple-negative breast cancers, metformin has been shown to suppress the metastasis-associated protein and breast CSC marker CD24 [139], suggesting a therapeutic role for metformin-based regimens in the clinical management of highly metastatic subgroups of triple-negative/basal-like breast carcinomas naturally enriched with CD24-positive tumor-initiating cells [140-142].

### Mechanisms of metformin cancer prevention (II): Epithelial-to-mesenchymal transition (EMT) and microRNAs

**Figure 2:**
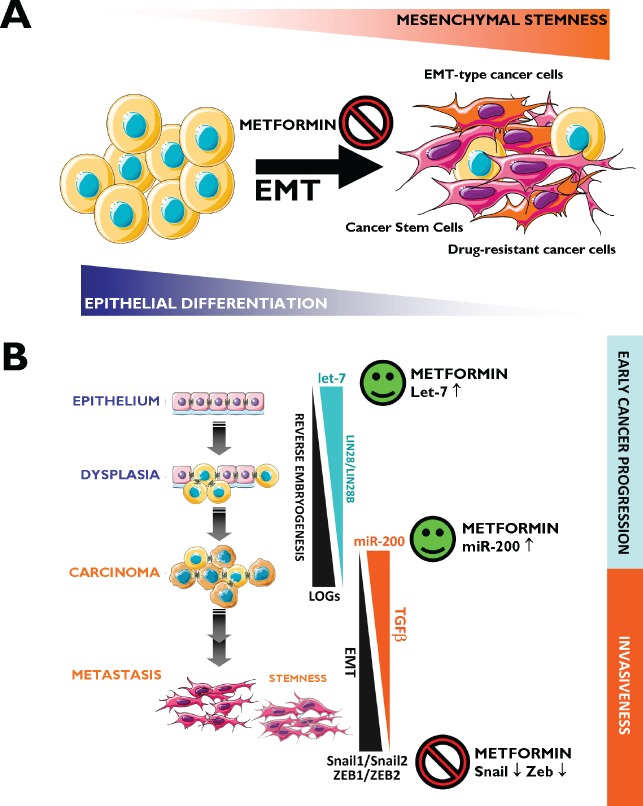
Metformin: A guardian of EMT and micro(mi)RNA-regulated stemness and cancer progression A. EMT Induction of the EMT transdifferentiation program in cancer cells results in the acquisition of invasive and metastatic properties. The emergence of CSCs also occurs in part as a result of EMT. In addition, EMT of tumor cells contributes to drug resistance. Figure depicts how metformin's ability to inhibit the EMT transdifferentiation program may represent a therapeutic strategy to clinically overcome chemotherapy refractoriness in CSCs-enriched invasive/metastastic carcinomas. B. Two evolutionary conserved families of miRNAs, let-7 and miR-200, regulate pivotal differentiation processes during development. On the one hand, loss of let-7 in cancer triggers reverse embryogenesis and dedifferentiation phenomena. On the other hand, miR-200 has been identified as a powerful regulator of the EMT process. The figure depicts how deregulation of let-7 and miRNA-200 during carcinogenesis could each contribute to tumor progression, one by controlling let-7 regulated oncofetal genes (LOGs) and, therefore, stem cell maintenance, and the other by regulating EMT and, therefore, the generation of migrating CSCs. Obviously, crosstalk exists between loss of let-7 –that results in reverse embryogenesis and dedifferentiation- and miR-200-regulated EMT –that results in up-regulation of a number of stem cell markers-. Metformin, through its ability to potentiate the expression of let-7a [153] and miR-200 (unpublished observations) and to prevent the overexpression of classical EMT markers such as ZEB, TWIST and SLUG (SNAIL2) [149], may function as an efficient molecular guardian against cancer progression and/or tumor recurrence after treatment.

The EMT has been established as a mechanism for the acquisition of stem cell characteristics (self-renewal and tumor initiation) [143-147]. Differentiated cancer cells lose their polarity, thus acquiring the properties of mesenchymal mobility, which allows a cell to leave the epithelial layer through the basal lamina and reach the bloodstream, thus permitting metastatic progression. Cancer-associated EMT cells lose epithelial characteristics, such as the expression of E-cadherin, while acquiring *de novo* expression of mesenchymal-associated genes. In breast carcinomas, these cells express the CD44^+^/CD24^−/low^ mesenchymal immunophenotype on the cell surface [148]. In highly metastatic basal-like MDA-MB-231 breast cancer cells, our group has shown that metformin treatment dynamically suppresses the CD44^+^/CD24^−/low^ CSC phenotype via transcriptional repression of the EMT machinery including Transforming Growth Factor-β1, ZEB, TWIST and SLUG (SNAIL2) [149]. Metformin-mediated regulation of E-cadherin can efficiently prevent the TGFβ-induced conversion of epithelial cells into migratory mesenchymal cells [150]. This treatment maintains the location of E-cadherin at sites of cell-cell contact and prevents the cellular changes (morphology and size) associated with a mesenchymal status. Therefore, metformin treatment could be a useful strategy to impede the formation of migrating CSCs [120] (Fig. 2A). We have recently proposed that metformin can alter micro(mi)RNA-regulated cell differentiation to prevent the invasion of human carcinomas. Invasion/metastasis of epithelial carcinomas including breast cancer can be viewed as a miRNA lethal-7 (let-7)-regulated continuum of progressive dedifferentiation (i.e., EMT) with a cell at the endpoint that has stem cell-like properties [151, 152]. As such, metformin's ability to upregulate let-7 expression in premalignant cells may efficiently push them to become less “embryonic” (i.e., mesenchymal stem-like cells) and more “normal” (i.e., non-stem differentiated epithelial cells). This could block the dynamic nature of cellular transformation and CSC formation in response not only to oncogenes but also to the local microenvironment [120, 153] (Fig. 2B).

## MECHANISMS OF METFORMIN IN CANCER PREVENTION (III): CELLULAR SENESCENCE

Studies of human cancer tissues and cancer-prone mice argue strongly that cellular senescence is an important physiological mechanism in protecting cells against malignant transformation [154-156]. Senescent cells can be found abundantly in intraepithelial premalignant lesions, whereas senescent cells are scarce in invasive, life-threatening metastatic carcinomas, supporting the notion that cellular senescence suppresses cancer *in vivo*. Dismantling the senescence response (e.g., via inactivation of the tumor-suppressor p53) causes a significant acceleration in the development of human tumors, whereas senescence in established malignant states is associated with tumor regression [157]. Senescence-inducing stressors such as oxidative damage, DNA damage and/or oncogenes normally trigger senescence at the premalignant tumor stage [158]. In agreement with an active role of cellular senescence in cancer prevention, the subsequent invasion of premalignant lesions almost inevitably involves one or more events that inhibit or impair the senescence pathway. For instance, the convergence of EMT-driven acquisition of stem cell characteristics with enhanced autophagy in response to bioenergetic stresses may turn premalignant phenotypes into tumor-initiating cells that bypass metabolic stress- and oncogene-induced cellular senescence [120, 125, 159-161]. Metformin's ability to enhance senescence in established premalignant disease or to trigger cellular senescence in fully malignant invasive disease is an unexplored mechanism that may explain metformin-mediated cancer prevention and treatment.

### Mechanisms of metformin-induced cellular senescence (I): Enhanced DNA Damage Response (DDR)-like signaling

In recent years, evidence has emerged that DNA Damage Response (DDR) is one of the earliest molecular events that impedes the multistep progression of human epithelial carcinomas to invasive malignancy. DNA damage can be due to a variety of factors such as telomere dysfunction and oncogene-induced “replication stress” [162-164]. Accordingly, there is a strong selective pressure for mutation in DDR components because activation of DNA damage checkpoints acts as the innate barrier against invasion/metastasis of tumors [162, 165]. Because the DDR is a major component of the innate tumor suppressor barrier in early human tumorigenesis, selective activation of DDR surveillance mechanisms may therefore directly contribute to metformin's cancer preventive effects. It would be interesting to test whether metformin can significantly increase senescence in premalignant lesions of the skin, the lung, the pancreas or the breast. Epithelial cells within premalignant breast lesions with markers of senescence maintain an intact response to cellular stress and are less likely to develop subsequent tumor events. In other words, the presence of functional pro-senescence mechanisms is the most accurate predictor of recurrence and progression of premalignant lesions *in situ* (e.g. in Ductal carcinoma *in situ* [DCIS] of the breast) to invasive basal-like breast carcinomas [166]. This evidence is valuable pre-clinical framework for pro-senescence metformin-based anti-breast cancer therapies, which could be evaluated in DCIS xenografts before and during the spontaneous transition to invasive breast cancer lesions [167-169]. As such, we are currently assessing whether cancer risk with metformin treatment is related to its ability to activate DNA damage-like signaling that induces specific senescence-like growth inhibition of premalignant or malignant cells without altering the normal function of non-neoplastic tissues.

Many tumor cells retain the ability to senesce in response to DNA-damaging chemotherapeutic agents in culture and *in vivo*. Because of this, it is tempting to suggest that, in the context of DDR, metformin-enhanced cellular senescence may underlie metformin's ability to increase the rate of pCR in neoadjuvant chemotherapy in diabetic patients with breast cancer [33] and to promote tumor regression and prevent relapse when combined with suboptimal doses of chemotherapy in animal models [102]. In primary murine embryonic fibroblasts from wild-type (p53^+/+^) mice, we recently tested whether metformin can regulate the senescence-like growth inhibition induced by doxorubicin, a DNA-damaging drug that induces cell senescence at concentrations significantly lower than those required for inducing apoptosis. Exposure to metformin increased the senescent subpopulations in control (untreated) cells and synergistically increased the cell senescence in response to doxorubicin-induced DNA damage [161]. It would also be relevant to evaluate whether metformin facilitates the “accelerated senescence” triggered in normal cells by the expression of mutated, transforming versions of oncogenes (e.g., *Ras* or *Raf*) and by some other forms of supraphysiological mitogenic signaling irrespective of senescence-inhibiting adaptations (e.g., inactivation of p53) [170-172]. Proliferative invasive cancer cells with activated oncogenes acquire instrumental mechanisms to suppress senescence in early stages of cancer pathogenesis (e.g., in *in situ* lesions); organisms in which cells fail to undergo senescence die prematurely of cancer [173]. Therefore, activating the program of senescence in tumor cells is an attractive approach to cancer treatment [157, 174] and may help to explain the differential impact of metformin on cancer incidence in non-prone and cancer-prone animal models and perhaps also in cancer-prone individuals. It remains to be elucidated, however, whether metformin's ability to strongly activate the ATM/Chk2-regulated DDR checkpoint [62] is a critical event that prevents neoplastic epithelium to progress unimpeded into invasive cancer in individuals without type 2 diabetes.

### Mechanisms of metformin-induced cellular senescence (II): Senescence bioenergetics in CSCs

It now appears that metformin can be added to the growing list of agents that have potent cancer chemopreventive properties by activating DDR signaling. Ongoing experiments in our laboratory have shown that chronic exposure to metformin drastically reduces the lifespan of non-transformed human fibroblasts by accelerating replicative cellular senescence (unpublished observations). In the presence of a constant mitogenic input, metformin treatment promotes an inappropriate culture environment that determines a new threshold level of negative signals rapidly surpassed and caused accelerated stress-induced senescence. Because culture conditions induce DNA damage in cultured human fibroblasts, metformin-accelerated replicative senescence may mostly rely on the ability of metformin to establish a stronger DDR-dependent cell cycle arrest. Alternatively, a lower threshold for stress-induced senescence due to metformin can be explained in terms of metformin-regulated energy metabolism. The most widely accepted interpretation for the biological function of cellular senescence is that it serves as a mechanism for restricting cancer progression. Based on this, escaping from cellular senescence and becoming immortal constitutes an additional step in oncogenesis that most tumors require for their ongoing proliferation [175]. Recent studies have suggested that the accumulation of reactive oxygen species (ROS) and oxidative damage are commonly involved in culture stress- or oncogene-induced senescence. Because increasing accumulation of ROS is observed during replicative senescence (i.e., the replicative potential of both murine and human fibroblasts is significantly higher under low oxygen), the ability of immortalized cells including embryonic stem cells, induced pluripotent stem cells (iPSCs) and CSCs to buffer oxidative stress may be pivotal for explaining their immortality [176-179]. Early studies by Warburg [180] found that most cancer cells metabolize glucose by enhanced glycolysis even in the presence of ample oxygen, despite the fact that this generates ATP less efficiently than the aerobic processes of respiration and oxidative phosphorylation (OXPHOS), which mainly occur in mitochondria. Glycolysis, which produces lactate from pyruvate, occurs predominantly in the cytoplasm and generates ATP more rapidly than respiration, which might offer a selective advantage to rapidly growing tumor cells. Thus, enhanced glycolysis even under 20% oxygen culture conditions is a characteristic property of most cancer cells and is known as the Warburg effect.

**Figure 3:**
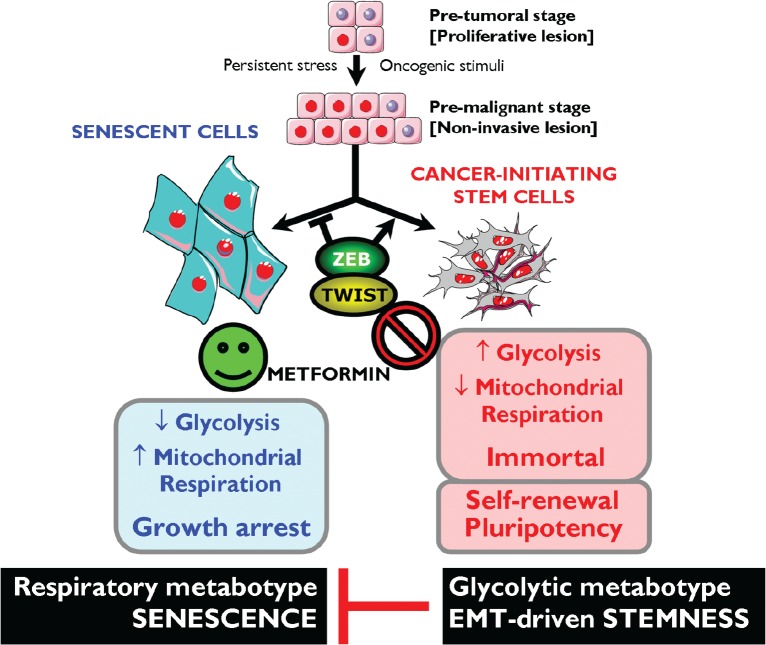
Metformin-targeted EMT and tumor metabolism: Novel strategy against CSCs *Top.* Oncogenic stimuli can either induce senescence or EMT, depending on the cellular and microenvironmental context. Conversely, EMT-inducing transcription factors can simultaneously suppress oncogene-induced senescence (OIS)-like responses and induce an EMT, both phenomena contributing to malignant progression because EMT generates migrating CSCs by directly linking enhanced cellular motility with the maintenance of tumor-initiating (stemness) capacity. *Bottom.* Rather than constituting a feature of malignancy *per se*, enhanced aerobic glycolysis and shifts in cellular metabolism away from mitochondrial respiration are intimately linked to malignancy at the level of CSCs. Enhanced glycolysis may play a causal role in the immortality of CSCs by protecting them from the senescent effects of mitochondrial respiration-induced oxidative stress. Metformin's ability to concomitantly attenuate the anti-senescence effects of both the EMT program and the ATP-generating glycolytic metabotype-the Warburg effect- may result in a phenotypic shift that impedes cancer oncogenesis by down-regulating self-renewal and proliferation of CSCs.

Although there have been limited mechanistic insights into the relationship between the Warburg effect with the well-characterized molecular and genetic events of cellular immortalization, we are beginning to accumulate evidence suggesting that the two phenomena are linked [176-179]. First, the glycolytic flux declines during senescence both in murine and human fibroblasts, while the expression of glycolytic enzymes can modulate cellular lifespan in mouse embryonic fibroblasts (MEFs) [176, 181]. Second, enhanced glycolysis can protect cells from oxidative stress and, consequently, avoid senescence triggered by oxidative stress [176]. Third, mouse embryonic stem cells, immortalized MEFs and iPSCs have surprisingly low rates of mitochondrial O_2_ consumption accompanied by a high glycolytic rate. Indeed, the *a priori* energetic infrastructure of somatic cells appears to be a crucial molecular feature for pluripotency because enhancing a bioenergetic shift from somatic oxidative mitochondria toward an alternative ATP-generating glycolytic process maximizes the efficiency of somatic reprogramming to pluripotency [179, 182]. Fourth, enhanced glycolysis plays an important role in the proliferative potential of stem cells; accordingly, they are hypersensitive to glycolytic inhibition and, once differentiated, they stop proliferation and drastically reduce their glycolytic flux. Altogether these data support the hypothesis that enhanced glycolysis actively protects cells from senescence induced by oxidative stress, a metabolic protection that might causally contribute to the maintenance of the self-renewal capacity of stem cells. The enhanced glycolysis of the Warburg effect is a crucial metabolic feature that helps bypass senescence, and this may provide indirect evidence that metformin's primary target is the immortalizing step during tumorigenesis (Fig. 3). During immortalization, several biological events are required beyond bypassing senescence such as growth factor independence, evasion of apoptosis, anti-growth signals, etc. If enhanced glycolysis is necessary and sufficient to enable indefinite proliferation (i.e., immortalization) very early during multi-step carcinogenesis *in vivo*, then metformin's ability to inhibit the glucose flux while simultaneously stimulating the lactate/pyruvate flux and mitochondrial biogenesis must cause ATP depletion accompanied by a drastic increase in cellular AMP, which is expected to induce premature senescence [183].

### Metformin and cancer prevention: Pre-clinical evidence

Over the next 5 to 10 years, the results of ongoing and planned metformin-based cancer studies will become available. Although it is anticipated that they will provide clinically relevant information regarding the actual effect of metformin on risk and outcomes in many common human cancers, pre-clinical studies can still provide crucial information on mechanisms of metformin's anticancer activity. These data could potentially facilitate the use of metformin as a novel agent for targeted cancer prevention. Studies examining the effect of metformin on colorectal carcinogenesis in chemically-induced animal models have demonstrated that metformin treatment efficiently suppresses azoxymethane-induced colorectal aberrant crypt foci via the inhibition of the mTOR pathway and through the activation of AMPK [184]. Based on these data, Hosono et al. [185] recently conducted a pilot clinical trial providing evidence that short-term, low-dose metformin (250 mg once daily for 1 month versus the typical 500 mg three times daily in type 2 diabetes) safely and directly suppresses both colorectal epithelial proliferation and aberrant crypt formation, an endoscopic surrogate marker of colorectal cancer, in prospectively randomized nondiabetic patients [185]. The gastrointestinal tract may be a special case where metformin appears to act locally from the lumen following oral administration; this raises the question of whether one could expect more enhanced benefits by achieving more continuous exposure to metformin (e.g., using the low-release metformin preparations developed for dosing convenience). This first reported trial demonstrated the potential for metformin in the chemoprevention of colorectal cancer. These findings were extended to lung tumors in the tobacco-specific carcinogen 4-(methylnitrosamino)-1-(3-pyridil)-1-butanone (NNK)-induced lung cancer mouse model. Using this model, Memmott et al. [186] recently demonstrated that treatment with high-dose metformin remarkably decreased tumor burden (~70%) without affecting tumor incidence, providing strong rationale for clinical prevention trials for lung cancer in heavy smokers. While there was no evidence of metformin-induced activation of AMPK in lung tumors, metformin led to decreased levels of circulating insulin and IGF as well as decreased phosphorylation of IGF-IR, AKT and mTOR in tumor tissue. Thus, the profound effects on tumor growth may have been a consequence of perturbation of glucose homeostasis and hormone levels leading to the indirect inhibition of mTOR by decreasing activation of IR/IGF-IR and AKT upstream of mTOR. A completely different picture was observed in a study with chemically induced mammary cancer in female Sprague-Dawley rats. Zhu et al. [187] reported that while a dosing regimen of 1.0%/0.25% metformin was capable of reducing palpable mammary carcinoma incidence, multiplicity and tumor burden and prolonged latency, lower doses of metformin failed to inhibit carcinogenesis despite reducing plasma insulin. Notably, metformin appeared to offer protection against new tumor occurrence following release from the combined treatment of metformin with dietary energy restriction. Because flow cytometry analyses indicated the presence of tumor-initiating cells in chemically induced mammary carcinomas, these findings support the hypothesis that metformin may be an effective component of multi-agent interventions against CSCs [187].

## METFORMIN: MULTI-FACETED PROTECTION AGAINST CANCER

**Figure 4:**
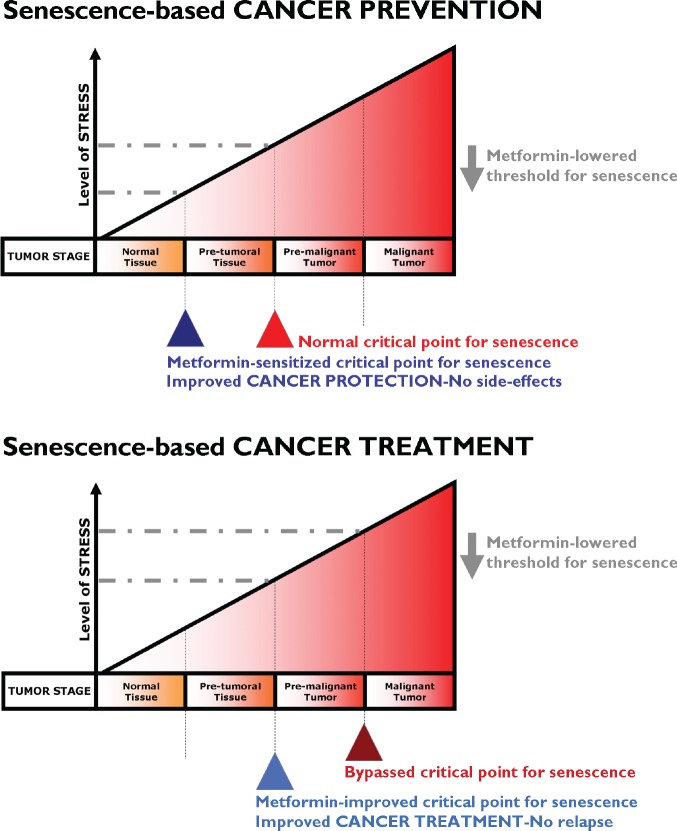
Metformin lowered threshold for senescence: Better protection and treatment against cancer With increasing levels of senescence-inducing stress (e.g., oncogenes, DNA damage, oxidative damage), tumor development goes through three stages namely pre-tumoral, pre-malignant and malignant tumor stages. The stressors normally reach sufficient intensity to trigger senescence only at the pre-malignant tumor stage. If the critical point for triggering senescence can be lowered by metformin by promoting DDR-like signals and/or impeding anti-senescence phenotypes (e.g., EMT, glycolytic metabotypes) in pre-tumoral (*top*) or tumoral (*bottom*) tissues, metformin treatment could translate into better protection against cancer (i.e., metformin's cancer prevention modality, *top*) and could impede progression to advanced and metastatic disease (i.e., metformin's cancer treatment modality, *bottom*). Our current ability to identify pre-malignant lesions has the potential to allow their early detection and treatment with metformin as a pro-senescence modality. In a neoadjuvant setting, metformin-induced senescence may reduce tumor growth and trigger the immune system to clear senescent cells, contributing to the reduction of tumor burden obtained with traditional chemotherapeutic and radiottherapeutic protocols. Metformin-based pro-senescence approaches may be also advantageous in the adjuvant setting, as it may have the ability to reduce the statistical risk of relapse from occult disease (e.g., residual disease in lymph nodes or systemic micrometastasis) that could arise from remaining quiescent CSCs.

We have reviewed the historic, epidemiological, pre-clinical and clinical studies in which the anti-diabetic biguanide metformin has suggested its unexpected use in oncology. It is unknown whether metformin would provide protective effects in people without diabetes, and we are still lacking additional physiologically relevant experimental models to elucidate whether the effects are via direct suppression of mTOR signaling in malignant (subclinical) or premalignant cells and/or by decreasing the levels of circulating hormones (which may promote the growth of malignant cells). However, a number of clinical trials examining the use of metformin as a cancer therapy are currently underway in prostate, breast, endometrial, and pancreatic cancer patients [188-194]. As auspiciously suggested by Dowling et al. [195], “the initiation of new, focused clinical trials containing strong correlative science components will be crucial in understanding the effects of the drug on a range of cancer patients (*including non-diabetic patients*) and the identification of biomarkers that predict metformin benefit and response to therapy.” Chemoprevention studies with metformin should ideally target patients with a high risk of developing cancers, such as those with premalignant conditions (e.g., DCIS of the breast, atypical adenomatous hyperplasia of the lung) or those with a high risk of disease recurrence (e.g., in the adjuvant setting) (Fig. 4).

If metformin therapy creates an intrinsic barrier against tumorigenesis by lowering the threshold for stress-induced senescence, metformin therapeutic strategies aimed to enhance senescence may be pivotal for therapeutic intervention of cancer. To test the hypothesis that metformin therapy is a senescence-based cancer prevention strategy (Fig. 4, *top*), we suggest the following study in women with DCIS lesions. Given that DCIS lesions contain pre-existing carcinoma precursor cells, it would be of interest to evaluate whether metformin use in nondiabetic women reduces the expected progression rate (12-15%) of DCIS lesions to invasive breast cancer. Taking advantage of previously used trial strategies for tamoxifen-based neoadjuvant therapy of DCIS [196], it might be reasonable to start a clinical trial for metformin-based neoadjuvant therapy of DCIS in which metformin is administered after diagnosis by a primary biopsy but before the commencement of standard-of-cancer surgical therapy. New contralateral tumors in women with breast cancer might be useful as a model for secondary prevention, similar to tamoxifen [197]. If valid, such a model would facilitate the testing of metformin therapy as a senescence-based cancer treatment strategy (Fig. 4, *bottom*). Taking advantage of previous trials on the anti-CSC activity of targeted drugs in a neoadjuvant setting [198, 199], paired core biopsies could be obtained from patients before and after treatment with metformin regimens. Cell populations isolated from biopsy samples taken before and after metformin-based therapy could be assayed for tumor-initiating cells by measuring their ability to form mammospheres *in vivo*, a widely accepted indicator of self-renewal. In parallel, the presence of senescent cells before and after metformin-based therapy could be detected by the classical test for SA-β-Gal activity using X-Gal (5-bromo-4-chloro-3-indolyl-β-D-galactoside) [200]. As suggested by Nardella et al. [157], this type of analysis may be readily performed on tumor biopsy samples, although it should be noted that biopsy samples should be frozen for optimal senescence analysis [201]. Immunohistochemical analysis of traditional senescence effectors (e.g., upregulation of p53, INK4A, p21 and p27) can also enable the detection of metformin's ability to induce senescence as part of its therapeutic effects.

Extensive clinical experience with metformin coupled with the preclinical rationale, potential mechanisms of metformin's anti-cancer effects and its modest toxicity have sped up the timeline of oncology drug development (Fig. 1). Indeed, metformin exemplifies how systems biology strategies for repositioning regulatory agency (FDA/EMEA)-approved drugs may accelerate our ability to prevent and/or treat cancer in a multifaceted manner, including elimination of CSCs. We anticipate the translational impact that metformin will have as a valuable oncology drug due to its ability to arrest carcinomas at their non-invasive, premalignant stages. Current [107, 193, 202] and future clinical trials will elucidate whether metformin has the potential to be used in both preventive and treatment settings as an adjuvant to current cancer therapeutics.
